# Review of the genus *Fibuloides* Kuznetsov in China (Lepidoptera, Tortricidae, Olethreutinae)

**DOI:** 10.3897/zookeys.81.833

**Published:** 2011-02-18

**Authors:** Aihuan Zhang, Houhun Li

**Affiliations:** 1 College of Life Sciences, Nankai University, Tianjin 300071, P. R. China; 2 Beijing Key Laboratory of New Technology in Agricultural Application, College of Plant Science and Technology, Beijing University of Agriculture, Beijing 102206, P. R. China

**Keywords:** Lepidoptera, Tortricidae, *Fibuloides*, new species, new combination, synonym, China

## Abstract

Species of the genus Fibuloides Kuznetsov that occur in China are reviewed. Fibuloides trapezoidea, **sp. n.**is described as new; Fibuloides levatana (Kuznetsov) and Fibuloides modificana Kuznetsov are newly recorded for China; Acroclita nigrovenana Kuznetsov, **syn. n.** is considered as a synonym of Fibuloides corinthia (Meyrick); and Eucoenogenes elongata Zhang & Li and Eucoenogenes wuyiensis Zhang & Li are transferred to Fibuloides, resulting in two new combinations. A key to the nine Chinese species of Fibuloides is given.

## Introduction

Fibuloides was proposed by Kuznetsov (1997b) with Fibuloides modificana Kuznetsov, 1997 from South Vietnam as the type species. The characters of Fibuloides given by Kuznetsov are as follows: the costal fold narrow in male; the forewing with R_4_ and R_5_ stalked, R_3_ with base close to this stem; CuA1 strongly curved and originating from near base of M3; hindwing with M3 and CuA1 stalked; and the valva extremely modified, with an unusually long, sclerotized process originating from the apex of the sacculus. Brown (2005) included one species, Fibuloides modificana, in the world catalogue of Tortricidae.

Horak (2006) described Fibuloides phycitipalpiaHorak, 2006 and Fibuloides minuta Horak, 2006 from Queensland and New South Wales, Australia, and transferred seventeen species to the genus. She pointed out that males of Fibuloides usually have a notch at the base of the flagellum, bear modified fringe scales along the anal margin of the hindwing or a pencil of long hairscales from its base, have transverse bands of modified scaling dorsally on the abdomen, and the weak lateral arms of the gnathos from below middle of the tegumen end in two sclerotized vertically rising bands. Pinkaew (2008) described Fibuloides khaonanensis from Thailandandtransferred Eucoenogenes bicucullus Pinkaew, 2005 and Eucoenogenes vaneeaefPinkaew, 2005 to Fibuloides.

Currently the genus includes 23 species distributed in the Palaearctic, Oriental and Australian regions (Brown 2005; Horak 2006; Pinkaew 2008; Baixeras et al. 2009). Prior to this study, five species were recorded from China (Kawabe et al. 1992; Liu and Li 2002; Zhang and Li 2005). In the present paper, we describe one new species, record two species new for the Chinese fauna, transfer two species from Eucoenogenes to Fibuloides, and propose Acroclita nigrovenana Kuznetsov, 1988 from North Vietnam as a synonym of Fibuloides corinthia (Meyrick, 1912) described from Sri Lanka. A key to the Chinese species is provided based on the male genitalia of the examined specimens.

## Material and methods

This study is based on the examination of specimens collected using light traps in the forests and mountains, mainly from the southern part of China. The terminology for the forewing pattern follows Brown and Powell (1991) as refined by Baixeras (2002). Methods of genitalia dissection follow Li (2002). Unless indicated otherwise, all the specimens examined, including the types, are deposited in the Insects Collection, College of Life Sciences, Nankai University, Tianjin, China.

Abbreviations

TLType locality;

BMNHThe Natural History Museum, London, UK;

ZMASZoological Institute, Russian Academy of Sciences, St. Petersburg (Leningrad), Russia;

NKUMInsects Collections, College of Life Sciences, Nankai University, Tianjin, China;

USNMNational Museum of Natural History, Washington, D. C., USA.

## Taxonomic accounts

### Genus Fibuloides

Kuznetsov, 1997

Fibuloides Kuznetsov, 1997: 810. Type species: *Fibuloides modificana*Kuznetsov, 1997: 810, by original designation.

#### Key to Chinese species of Fibuloides based on male genitalia

**Table:** 

1	Uncus blunt apically	2
–	Uncus pointed apically	4
2	Sacculus with a digitate process, neck of valva with two enlarged, flattened bristles	Fibuloides japonica
–	Sacculus without digitate process, neck of valva with one or more than three enlarged, flattened bristles	3
3	Uncus drooping, projected outward, socius upturned; neck of valva with five enlarged, flattened bristles on left side and four on right side, cucullus somewhat elliptic	Fibuloides elongata
–	Uncus not drooping, socius drooping, neck of valva with one enlarged, flattened bristle, cucullus somewhat trapezoidal	Fibuloides modificana
4	Neck of valva without enlarged, flattened bristles	5
–	Neck of valva with one or more enlarged, flattened bristles	7
5	Cucullus bipartite, nearly ovate, or nearly trapezoidal	6
–	Cucullus very long and narrow, with long point on end	Fibuloides levatana
6	Cucullus nearly trapezoidal, uncus with tips straight and parallel	Fibuloides trapezoidea
–	Cucullus bipartite, nearly ovate, uncus with tips bent outward	Fibuloides corinthia
7	Socius triangular, angle of sacculus indistinct	Fibuloides wuyiensis
–	Socius lobe-shaped, angle of sacculus obtuse	8
8	Cucullus nearly triangular; socius broad and short, about twice as long as wide	Fibuloides aestuosa
–	Cucullus ovate; socius slender, about three times as long as wide	Fibuloides cyanopsis

#### Fibuloides trapezoidea

Zhang & Li sp. n.

urn:lsid:zoobank.org:act:EA201525-B594-47F4-A928-6C254A1B37C6

Figs 1 10 

##### Type material.

Holotype ♂ **–** **China, Guizhou Province:** Chishui (28°34'N, 105°42'E), 390 m, 30.V.2000, coll. Yanli Du, genitalia slide no. YHL04481; Paratype **–** 1 ♂, 27.V.2000, other same data as for holotype, genitalia slide No. ZAH10019.

##### Diagnosis.

This species is similar to Fibuloides cyanopsis(Meyrick, 1912) in the shape of uncus and socius, but can be distinguished by the trapezioidal cucullus and the absence of the enlarged, flattened bristles on the neck of valva. In Fibuloides cyanopsis the cucullus is ovate and the neck of valva has two or more short enlarged, flattened bristles on its ventral side.

##### Description.

**Figures 1–9.**
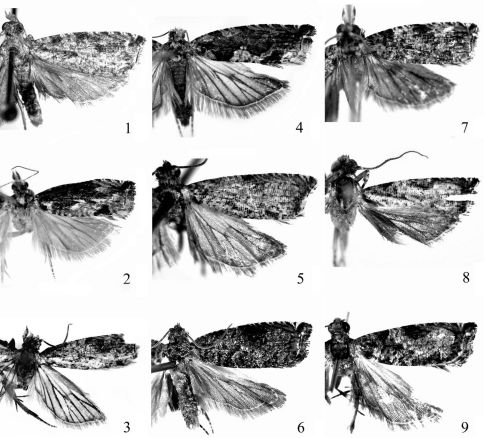
Adults of Fibuloides spp. **1** Fibuloides trapezoidea Zhang & Li, sp. n., ♂ **2** Fibuloides aestuosa (Meyrick), ♂ **3** Fibuloides corinthia (Meyrick), ♂ **4** Fibuloides cyanopsis (Meyrick), ♀ **5** Fibuloides elongata (Zhang & Li), ♂ **6** Fibuloides japonica (Kawabe), ♂ **7** Fibuloides levatana (Kuznetsov), ♂ **8** Fibuloides modificana Kuznetsov, ♂ **9** Fibuloides wuyiensis (Zhang & Li), ♂.

Adult (Fig. 1). Forewing length 6.5 mm. **Head:** Vertex with gray scales; frons white. Antenna light brown. Labial palpus slender, gray intermixed with brown, third segment porrect. **Thorax:** Thorax and tegula gray intermixed with light brown. Forewing elongate triangular, with ground color dark gray; basal patch extending from costal 1/4 to 1/3 of dorsum, protrudent in middle on outer side; median fascia short and broad, extending from costal half, terminated at end of cell; ocellus nearly quadrate, containing some short brown striae; apex brown, protrudent; termen slightly concave below apex, bordered by brown scales; costa with nine pairs of strigulae from base to apex, each pair of strigulae with a short brown stria extending obliquely; first to fourth pairs between base of wing and the point where Sc meets costa, broad brown patch lying between second and third pairs; fifth and sixth pairs between Sc and R_1_; distal three pairs distributed between pairs of veins R_1_–R_2_, R_2_–R_3_, R_3_–R_4_ respectively, separated from each other by dark brown scales; cilia gray mixed with brown. Hindwing and cilia gray. Legs gray, tarsi with brown rings.

**Figures 10–18.**
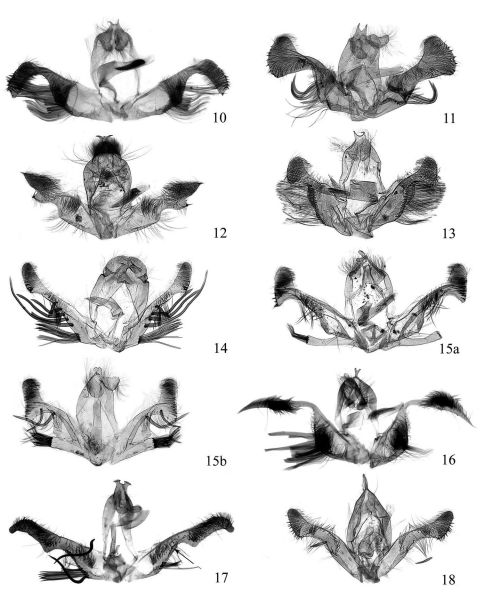
Male genitalia of Fibuloides spp. **10** Fibuloides trapezoidea Zhang & Li, sp. n., slide No. YHL04481 **11** Fibuloides aestuosa (Meyrick), slide No. ZAH03603 **12** Fibuloides corinthia (Meyrick), slide No. ZAH04036 **13** Fibuloides cyanopsis (Meyrick), slide No. ZAH03758 **14** Fibuloides elongata (Zhang & Li), slide No. ZAH03725 **15a–15b** Fibuloides japonica (Kawabe), slide Nos. **a**: ZAH03173, **b**: ZAH04296 **16** Fibuloides levatana (Kuznetsov), slide No. ZAH04299 **17** Fibuloides modificana Kuznetsov, slide No. LJM04401 **18** Fibuloides wuyiensis (Zhang & Li), slide No. ZAH04215.

##### Abdomen:

Male genitalia (Fig. 10). Uncus bifurcated from near base, produced into two slender, parallel and widely separated tips. Socius broad and short, about twice as long as wide, drooping, hairy, with round end. Valva broad at base; neck distinct, without enlarged, flattened bristles; sacculus about twice size of cucullus, with a line of flattened long bristles; cucullus trapezoidal, hairy, with marginal spines. Anellus closely surrounding basal 1/3 of aedeagus; aedeagus long tubular, cornuti consisting of a bunch of curved spines.

##### Female.

Unknown.

##### Distribution.

China (Guizhou).

##### Etymology.

The specific name is derived from Latin *trapezoideus* (= trapeziform), referring to the shape of the cucullus.

#### Fibuloides aestuosa

(Meyrick, 1912)

Figs 2 11 19

Spilonota aestuosa  Meyrick 1912: 854. Holotype ♂, TL: India, deposited in BMNH.
Acroclita ligyropis  Meyrick, 1937: 176; Clarke 1958: 267.
Eucoenogenes aestuosa  (Meyrick, 1912): Kuznetsov 1976: 12; Kawabe 1982, 1: 120, 2: 173; Razowski 1989: 256; Byun et al. 1998: 160; Razowski 1999: 446; Kuznetsov 2001: 402; Liu and Li 2002: 319.
Fibuloides aestuosa  (Meyrick, 1912): Horak 2006: 330.

##### Material examined.

**China,** **Sichuan Province:** 1 ♀, Baoxing County, 1600 m, 3.VIII.2004, coll. Yingdang Ren. **Zhejiang Province:** 2 ♂♂, 3 ♀♀, Mt. Tianmu, 350–1500 m, 18~20.VIII.1999, coll. Houhun Li *et al*. **Hubei Province:** 2 ♂♂, 3 ♀♀, Hefeng County, 1260 m, 15–16.VII.1999, coll. Houhun Li *et al*. **Henan Province:** 1 ♀, Song County, 1580 m, 23.VII.2002, coll. Xinpu Wang.

##### Host plants.

Fagaceae: Castanea mollissima Blume and Castanea cranata Sieb. *et* Zucc. (Kuzenetsov 2001; Liu and Li 2002).

##### Distribution.

China (Anhui, Henan, Hubei, Guangxi, Liaoning, Sichuan, Yunnan, Zhejiang), Korea, Japan, India, Bengal.

##### Remarks.

The number of thick flattened bristles below the neck of the valva in the male genitalia is variable (two or more).

#### Fibuloides corinthia

(Meyrick, 1912)

Figs 3 12

Acroclita corinthia Meyrick, 1912: 858; Diakonoff 1950: 277; Clarke 1958: 271; Kawabe et al. 1992: 108. Lectotype ♂, TL: Sri Lanka, deposited in BMNH.
Fibuloides corinthia  (Meyrick, 1912): Horak 2006: 330.
Acroclita nigrovenana  Kuznetsov, 1988: 88; Nasu 1993: 216;Kuznetsov 2001: 408. Holotype ♂, TL:Vietnam, deposited in ZMAS. **syn. nov.**Fibuloides nigrovenana (Kuznetsov, 1988): Horak 2006: 330.

##### Material examined.

**China, Yunnan Province:** 1 ♂, Yuanjiang County, 710 m, 28.IV.1995, coll. Guangyun Yan.

##### Host plant.

Sapindaceae: Litchi chinensis Sonn. (Kuznetsov 2001).

##### Distribution.

China (Yunnan, Taiwan); Sri Lanka; India.

##### Remarks.

The male uncus is produced into two sharp, outwardly bent tips, and the cucullus is nearly elliptic, ending in a spine, with a ventral process bearing a short distal spine.

Diakonoff (1950) designated the male lectotype and Clarke (1958) provided photographs of the adult and male genitalia. Horak (2006) transferred Acroclita corinthia and Acroclita nigrovenana to Fibuloides. We synonymize Fibuloides nigrovenana with *corinthia* based on the the study of the adult and the male genitalia. Though we were unable to locate the type of Fibuloides corintha in BMNH, the two species appear to be conspecific based on a comparison of the photo in Clarke (1958) and the illustration in Rose and Pooni (2005)of Acroclita corinthia with the photo of Acroclita nigrovenana from Japan in Nasu (1993) as well as with the adult and male genitalia of the Chinese specimen.

**Figures 19–21.**
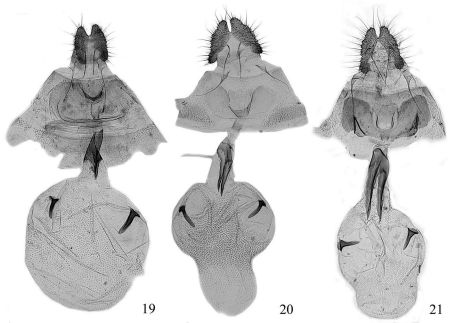
Female genitalia of Fibuloides spp. **19** Fibuloides aestuosa (Meyrick), slide No. ZAH03604 **20** Fibuloides cyanopsis (Meyrick), slide No. ZAH03757 **21** Fibuloides japonica (Kawabe), slide No. ZAH03645.

#### Fibuloides cyanopsis

(Meyrick, 1912)

Figs 4 13 20

Eucosoma cyanopsis  Meyrick, 1912: 866; Clarke 1958: 360. Holotype ♂, TL: India, deposited in BMNH.
Eucoenogenes cyanopsis  (Meyrick, 1912): Kuznetsov 1988: 81; Kawabe 1989: 52; Razowski 1999: 446; Kuznetsov 2001: 402.
Fibuloides cyanopsis  (Meyrick, 1912): Horak 2006: 330.
Eucosma melanochlaena  Meyrick, 1936: 611. Holotype ♂, TL: Indonesia, deposited in BMNH.

##### Material examined.

**China,** **Guangxi Zhuangzu Autonomous Region:** 1♂, 3 ♀♀, Shangsi County, 260–770 m, 1–3.IV.2002, coll. Shulian Hao and Huaijun Xue; 1 ♂, 3 ♀♀, Mt. Maoer, 550–1100 m, 19–20.IV.2002, coll. Shulian Hao and Huaijun Xue. **Guangdong Province:** 1 ♀, Lianzhou, 650 m, 23.VI.2004, coll. Dandan Zhang. **Guizhou Province:** 1 ♀, Jiangkou County, 1700 m, 29.VII.2001, coll. Houhun Li and Xinpu Wang.

##### Distribution.

China (Guangdong, Guangxi, Guizhou), Japan, Vietnam, Indonesia, India.

##### Remarks.

The cucullus of this species is ovate, and the neck of valva has two or three short enlarged, flattened bristles.

#### Fibuloides elongata

(Zhang & Li, 2005) comb. n.

Figs 5 14

Eucoenogenes elongata  Zhang & Li, 2005: 126. Holotype ♂, TL: China, deposited in NKUM.

##### Material examined.

Holotype ♂, **China, Yunnan Province:** Weishan County, 2200 m, 20.VII.2001, coll. Houhun Li and Xinpu Wang, genitalia slide no. ZAH03725.

##### Distribution.

China (Yunnan).

##### Remarks.

This species can be easily distinguished from its congeners by the drooping uncus with a blunt apex, and the narrow valva with the neck bearing five long, flattened flagellate bristles on the left side and four on the right side.

#### Fibuloides japonica

(Kawabe, 1978)

Figs 6 15a–15b 21

Eucoenogenes japonica  Kawabe, 1978: 185; Kawabe 1982, 1: 120, 2: 173; Byun et al. 1998: 160; Razowski 1999: 446; Kuznetosv 2001: 402; Liu and Li 2002: 319. Holotype ♂, TL: Japan, deposited in USNM.
Fibuloides japonica  (Kawabe, 1978): Horak 2006: 330.

##### Material examined.

**China, Shaanxi Province:** 2♂♂, Ningshan County, 880 m, 17.VI.1987, coll. Houhun Li; 9 ♂♂, 3 ♀♀,Yangxian County, 600–680 m, 17–18.IV.1995, coll. Hongjian Wang. **Henan Province:** 3 ♂♂, Luoshan County, 350 m, 2.V.2000, coll. Haili Yu; 2 ♂♂, Xinyang City, 700 m, 11.VII.1997, coll. Houhun Li; 1♂, Tongbai County, 300 m, 11.IX.2001, coll. Houhun Li and Ole Karsholt; 2 ♂♂, Chishui County, Guizhou Province, 240 m, 21–22.IX.2000, coll. Haili Yu. **Guizhou Province:** 1 ♂, Xishui County, 500 m, 25.IX.2000, coll. Haili Yu; 5 ♂♂, 1 ♀, Daozhen County, 1300 m, 20.VIII.2004, coll. Yunli Xiao. **Zhejiang Province:** 1 ♂, Mt. Tianmu, 650 m, 20.VIII.1999, coll. Houhun Li. **Hunan Province:** 1 ♀, Zhangjiajie, 650 m, 7.VIII.2001, coll. Houhun Li and Xinpu Wang. **Fujian Province:** 1 ♂, Nanping, 850 m, 23.IX.2002, coll. Xinpu Wang; 1 ♂, Jianning County, 350 m, 25.IX.2002, coll. Xinpu Wang. **Hubei Province:** 1 ♂, Hefeng County, 1260 m, 16.VII.1999, coll. Houhun Li.

##### Distribution.

China (Zhejiang, Anhui, Fujian, Henan, Hubei, Hunan, Sichuan, Guizhou, Shaanxi, Taiwan), Korea, Japan.

##### Remarks.

This species is distinguished by the digitate process on the sacculus in the male genitalia bearing either dense tufted bristles or five enlarged, flattened bristles distally.

##### Discussion.

In the examined specimens, the appearance of the adults and the female geniatlia are identical, but the male genitalia have two types (Figs 15a and 15b): in figure 15a, the relatively elongate uncus looks like a pair of long ears of a rabbit, and the slender digitate process of the sacculus bears five enlarged, flattened bristles distally; in figure 15b, the short uncus is emarginated posteriorly and somewhat heart-shaped, and the digitate process of the sacculus is relatively broad and bears dense tufted bristles distally. The two types of male genitlia might represent two different species, but in this paper we treat these differences as individual variations. We may confirm whether they are two species or just one species after a geographic analysis, which can be done when more specimens are available.

#### Fibuloides levatana

(Kuznetsov, 1997)

Figs 7 16

Eucoenogenes levatana  Kuznetsov, 1997: 197. Holotype ♂, TL: Vietnam, deposited in ZMAS.
Fibuloides levatana  (Kuznetsov, 1997): Horak 2006: 330.

##### Material examined.

**China, Zhejiang Province:** 1 ♂,Mt. Tianmu, 350 m, 20.VIII.1999, coll. Houhun Li et al. **Fujian Province:** 1 ♂, Yongtai County, 550 m, 18.IX.2002, coll. Xinpu Wang.

##### Distribution.

China (Zhejiang, Fujian), Vietnam.

##### Remarks.

This species can be easily distinguished by its Y-shaped uncus and the slender distally attenuate cucullus. It is new for China.

#### Fibuloides modificana

Kuznetsov, 1997

Fig. 8 17

Fibuloides modificana  Kuznetsov, 1997: 810. Holotype ♂, TL: Vietnam, deposited in ZMAS.

##### Material examined.

**China, Guangxi Zhuangzu Autonomous Region:** 1 ♂, Leye County, 665 m, 24.VII.2004, coll. Jiasheng Xu.

##### Distribution.

China (Guangxi), Vietnam.

##### Remarks.

The uncus of this species is broad and distally bifurcate, the valva has a long, sinuate, flattened bristle on the neck, and the cucullus is elongatesubrectangular and distally downcurved. In figure 17 the long flattened bristle is off the inserted hole which is located on the ventral side of the neck instead of on the angle of the sacculus (see arrow in fig. 17).It is new for China.

#### Fibuloides wuyiensis

(Zhang & Li, 2005) comb. n.

Figs 9 18

Eucoenogenes wuyiensis  Zhang & Li, 2005: 127. Holotype♂, TL: China, deposited in NKUM.

##### Material examined.

Holotype ♂, **China**, **Fujian Province:** Mt. Wuyi, 1000 m, 26.V.2004, coll. Haili Yu, genitalia slide no. ZAH04215; Paratype: 1 ♂, same data as for holotype.

##### Distribution.

China (Fujian).

##### Remarks.

This species is distinguishable from its congeners by the following characters: the uncus tips are slender and closely parallel; the socius is laterally triangular; and the neck of the valva has a short flattened bristle.

## Supplementary Material

XML Treatment for Fibuloides
